# Technical, Economic, and Sustainability Evaluation Integrated with PI System Monitoring: A Case Study on Different Process Alternatives for Acetone Production

**DOI:** 10.1021/acsomega.5c02293

**Published:** 2025-07-23

**Authors:** Matheus Vittori Ferreira, André Ferreira Young, José Carlos Costa da Silva Pinto

**Affiliations:** † Escola de Química, 28125 Universidade Federal do Rio de Janeiro, Av. Athos da Silveira Ramos, 149, Rio de Janeiro 21945-970, Brazil; ‡ Departamento de Engenharia Química e de Petróleo, 28110 Universidade Federal Fluminense, Rua Passo da Pátria, 156, Niterói 24220-900, Brazil

## Abstract

Process optimization, monitoring, and control play important roles in chemical plants. In an ever-changing environment related to climate concerns, reducing costs, keeping the product’s quality, producing more, and finding eco-friendly solutions are crucial to a company’s financial stability. Therefore, technical–economic techniques are often applied to verify the feasibility of those practices. Alternative scenarios on process configurations of acetone production via the dehydrogenation of isopropyl alcohol are the object of study of this work. A temperature optimization case study in the reactor was executed to evaluate the possibility of increasing the production. An economic analysis was performed for each scenario, comparing them and evaluating their attractiveness. To simulate as if they were a real and live plant, a PI System environment was developed, creating a dashboard for variables, key process indicators (KPIs), and process anomalies monitoring, and sending alerts if needed. The combined energy source change and temperature optimization scenario made the process economically feasible, increasing revenues by 5.2% and reducing production costs by 0.26%, achieving a net present value (NPV) of US$4.63 M (372% higher than the baseline). This work is a demonstration of how associating process data with a data historian and analyzer allows live testing, enhances safety, and deepens KPI monitoring.

## Graphical abstract

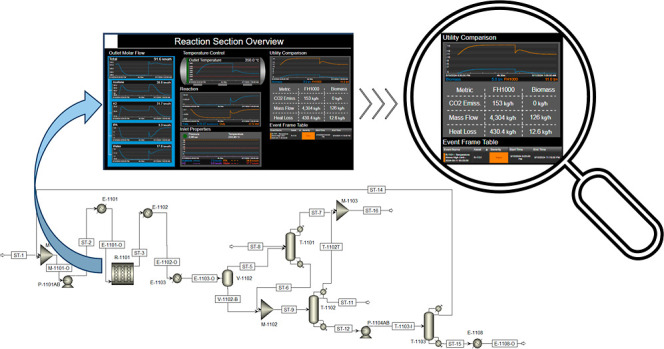

## Introduction

### Contextualization

Developing a project and building an industrial plant require several engineering calculations, technical–economic viability evaluations and projections, process yield analysis, product relevance analysis to society, and social and environmental potential impact identification. Nowadays, it is noted that commercial, governmental, and industrial organizations focus on improving their management strategies by applying policies toward sustainable development. Those policies lead companies to refine their technical specifications to obtain the desired products, aiming for quality enhancement while reducing costs and mitigating environmental effects. Smith^1^ and Seider^2^ highlight process simulation as a convenient strategy to quickly evaluate process modifications in terms of mass and energy balances, equipment design, and economic viability.

If one is willing to alter a project, simulating the proposed modifications can be beneficial, since it is possible to evaluate the consequences of changing pieces of equipment and flow rates without intervening in the real—operating—plant. On behalf of altering a project, reducing its costs, making it more environmentally friendly and more efficient with respect to utility usage, and looking for and testing other utility sources can be a viable solution.^3–7^ Prior to testing any change in the real process, a simulation must be executed to confirm the gains of the new process configuration. Therefore, simulated enhancement techniques must be evaluated in terms of economic viability and environmental sustainability. A financial analysis must be done in order to state the robustness and attractiveness of the project, allowing the companies to predict different scenarios considering economic factors.^8^ Additionally, quantitative and qualitative metrics must be considered to evaluate the environmental impact of those processes.

With the increasing need to reduce fossil fuel consumption and its greenhouse gas emissions, renewable fuel sources and biofuels gained more protagonism since they can be considered CO_2_ neutral.^9^ A strong candidate and subject of testing in the present work is biomass. It is composed of natural constituents or generated by animal and human food digestion. The biomass source influences its composition and characteristics, such as a higher heating value (HHV). Therefore, if that biofuel is economically attractive, it can be considered as an alternative for fossil resources like natural gas, a common energy source in chemical processes.^10–12^

However, choosing the best fuel to use, for example, requires some data and indicators that are important to the company, such as its price and associated CO_2_ emissions. Additionally, operators will take action to control the process properly, based on data. On the one hand, traditionally, plant personnel can only control the plant processes and variables and, moreover, monitor them while being on site, more specifically in the control room, but advances in data processing and transmission have made it possible to monitor processes and send actions to the operators to correct failures or deviations from elsewhere, also triggering alerts when the variables are not within the adequate ranges. The concept of Industry 4.0 ^13–16^ promoted innovative and advanced technologies capable of dealing with large volumes of process data, integrating process areas, optimizing production, prioritizing decision making, enhancing process efficiency and flexibility,^17–24^ and, most importantly, providing online availability of all kinds of data, which is only possible due to data historians and analyzers.^25–28^

In the academic field, data historians are commonly used as databases, a data pool to store and provide the necessary data to be used somewhere else, such as by a control tuning or machine learning application to calculate the best tuning parameters or to optimize process variables.^29–31^ Those values may be sent back to the data historian so they are available to the plant personnel to help them with decision-making. However, the true potential of data historians, such as key process indicator (KPI) calculation, trend and bottleneck analyses, process anomaly warning, and alarm generation, is not exploited due to lack of knowledge within companies’ employees. This is the gap that this work is meant to fill. Having all these features in just one place, hence the data historian and analyzer, without the need to open several applications or contact multiple people to seek information, would facilitate plant personnel’s work.

The importance of data historians and analyzers increased with the COVID-19 pandemic since more people were sent home to work and still needed to pass on instructions to plant operators, the few that were required on site. Worldwide, more than 1000 companies use PI System, which is “an edge-to-cloud data management system that provides trusted operational data for data-driven decision-making and digital transformation in a distributed enterprise”.^32,33^ The PI System is a largely used data historian, analyzer, and monitor, centralizing all data, making it available to all plant personnel with the proper credentials. Thus, companies can make smart and correct decisions based on data, apply business rules and calculations to data, and, most importantly, analyze data across time and monitor them even away from the site, which conventional control systems do not have the ability to do, even helping to discover bottlenecks in the process, along with enhancement, optimization, and cost-reduction opportunities, by analyzing more than one variable at a time. One example of a success case is the one from UC Davis, which practically decreased in half the energy-use intensity of the campus, saving US$15 million since 2009.^34^ It can also provide alerts, via e-mail or SMS, with the desired variables to raise awareness of process deviations and speed up the process correction or help with investigation. Process anomalies are therefore easily targeted and corrected, bringing all the auxiliary variables into context for the best solution.

This work has the objective of evaluating, through simulations in the software Aspen Plus v.12.1 and an integrated economic and sustainability analysis, possible scenarios for the acetone production process and of illustrating how plant personnel would monitor the process on a daily basis and be informed about its deviations using the PI System. The analysis aims to determine whether it is possible to obtain a more advantageous process with some modifications to the real and conventional process. The alternative scenarios mentioned consist of three process derivations: burning biomass as a fuel source for heating utility, optimizing the reactor’s temperature, and the combination of them both. Process simulation not only represents time and cost savings in this study but also presents the possibility to evaluate all the possible configurations at once, which might not be possible on site since the different technologies would hardly be available.^35,36^ Apart from process variables, other KPIs, such as economic and sustainability indicators, were also built into the PI System. The use of the PI System aims to demonstrate its capabilities as a powerful data historian, analyzer, and visualization tool to plant personnel. Therefore, changes to the process that reflect those metrics are rapidly noticed through a dashboard and may be evaluated by everyone in the company, from field to office employees. The contribution of this work is to demonstrate how a data historian can be coupled with a process simulator or real and live plant data and provide benefits to end-users, from operation to management, such as centralizing process data; closer monitoring and providing more details about process anomalies; calculating process KPIs; and evaluating different production and economic scenarios.

### Process Description

A detailed view of the conventional production process, depicted in Figure 1, is necessary to accurately comprehend the simulated steps and adaptations. The production process of acetone via dehydrogenation of isopropyl alcohol (IPA) is adapted from ref 37, trying to replicate the chosen pieces of equipment, constraints, and utilities. There are plants that operate according to this process, as stated by the reference work.^37^ In the conventional acetone production process, an IPA feed stream 1, at 25 °C and 1.01 bar, feeds a horizontal tank V-1101 at 51.96 kmol/h. A recycle stream 14, which comes from the second distillation tower T-1103 and contains 65.0 mol % IPA, feeds the same tank. Recirculation aims to make full use of the nonreacted reactants, thus enhancing the global process conversion. The tank’s outlet stream 2 is pressurized by a pump P-1101A/B to 2.30 bar and heated to 234 °C at the heat exchanger (HEX) E-1101, which has high-pressure steam (HPS) as hot fluid. This guarantees that the IPA stream will feed the vertical reactor R-1101—which contains 448 tubes of 2 in. diameter and 20 ft length, filled with catalyst—at the proper temperature for the occurrence of the reaction.

**Figure 1:**
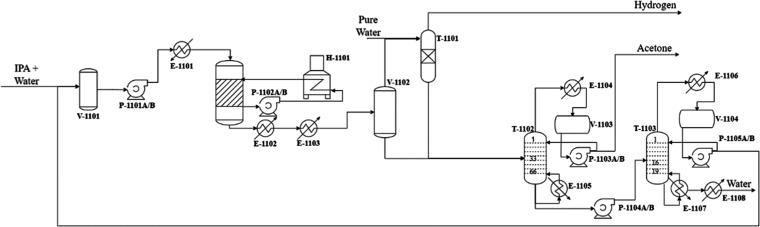
Conventional process flowsheet based on Turton.^37^

A furnace provides a hot saline solution at 407 °C and 2.66 bar to the reactor, arranging the necessary heat to keep the reaction medium at 350 °C, the optimal reaction temperature according to ref 37. The reactor’s product (stream 3) leaves the reactor at 350 °C, 1.91 bar, and 91.21 kmol/h. This stream contains 37.7 mol % acetone and is cooled down to facilitate the separation of the products and remaining reactants, according to thermodynamic equilibrium. Two HEXs are required to do it: E-1102 uses cooling water (CW), and E-1103 uses river water (CHILLED) as coolants.

Afterward, its components (IPA, hydrogen gas, and acetone) are separated in a flash vessel V-1102. The top product is sent to an absorption column T-1101, where it flows counterflow against pure water (stream 8). At the top of the column, hydrogen leaves and is stored, whereas water flows down to the bottom and joins the flash vessel’s bottom product to feed the 33rd stage of the first distillation column T-1102. This column has 66 stainless steel sieve plates spaced out by 18 in., one reboiler E-1105, and one partial condenser E-1104, both operating at 1.4 bar. Acetone is the distillate (stream 11), while the other remaining components make up the bottom product (stream 12), which flows to the next distillation column T-1103, feeding it at the 16th stage.

The second column has 19 stainless steel sieve plates spaced out by 24 in., one reboiler E-1107 and one total condenser E-1106, both operating at 1.4 bar. The bottom stream (stream 15) contains mostly water and is cooled to 45 °C at the HEX E-1108 prior to flowing to a disposal section. The distillate (stream 14) contains mostly IPA and is recycled at the beginning of the process. E-1105 and E-1107 use low-pressure steam (LPS) as a hot utility.

All process stream and utilities properties resulting from the studied routes are presented in Tables S1 and S2 of the Supporting Information.

### Biomass Usage

After searching for alternatives for the fuel source for steam generation, biomass was chosen to substitute for the natural gas consumption reported in the reference. However, its properties, such as a higher heating value,^7^ vary depending on its composition,^7^ which is affected by its source/species,^38–49^ place and manner of collection, storage and handling,^6,38,44,50,51^ processing and growing conditions,^5,40,42,43^ and contamination.^42,49,52^ Therefore, a standard biomass composition must be defined.

Some aspects must be taken into account when defining biomass.^7^ There is not a “universal dictionary” for biomass, and concepts are mistaken, leading to confusion in terminology, classification systems, and standards.^6,53^ Although there is a massive quantity of data, initiatives, and scientific articles regarding biomass, they are not consistent or cross-checked, resulting in misunderstandings. Identifying and characterizing biomass’ chemical and phase compositions is crucial to defining its properties. These can be achieved via proximate (fixed carbon, volatile matter, and ash yield), ultimate (C, O, H, S, and N), and ash (Si, Al, Fe, and others) analyses. Vassilev developed a reliable biomass database, containing 86 varieties of biomass, with their compositions and available analyses.^7^

As biomass will be the HEX’s and reactor’s energy source, one must estimate its HHV.^40,54^ Proximate and ultimate analyses results allow the correlation of HHV with biomass’ composition.^55–62^ A study was conducted by Sheng and Azevedo^63^ to test several correlations that were accurate for the reference databases used, but the models were inaccurate when exposed to other biomass samples. This occurred due to limited biomass samples and the method used in formula derivation. Therefore, they developed and statistically tested new correlations considering those analyses, with improved accuracy, creating quasi a “universal” correlation regarding the analysis type, covering more biomass samples than the ones used in their references. Their ultimate analysis’ correlation is more accurate and favorable to be used in engineering applications because only the composition of main elements matters, achieving more than 90% prediction accuracy with ±5% error.^63^ Therefore, this one was used in this work to estimate biomass’ HHV.

The aforementioned modifications were applied to the process. Inlet and outlet conditions and film coefficient were assumed the same for HPS and LPS, changing only their energy price and CO_2_ emission factor, since its source is now biomass. It is important to emphasize that not all types of biomass data were available, as related in the reference works.^7^ Therefore, using a standard/mean biomass was a strategic choice to have consistent data and values across the simulation. There was not a change of pieces of equipment but of their utility usage and utilities’ properties.

### Reactor Temperature Optimization

Although the biomass usage process seems promising for reducing operational costs, it does not affect production. So, we considered a scenario for enhancing production within the original configuration. For this, a sensitivity analysis has been made on some equipment units and indicated the reactor’s temperature as a good candidate for optimization.

The reactor’s temperature is crucial to the reaction thermodynamics and therefore influences product generation. By increasing it, the reaction equilibrium is shifted to the product’s side, as it is an endothermic reaction. Of course, the opposite occurs when it is decreased. It is not possible to increase the temperature infinitely and increase product formation due to safety, thermodynamics, and thermal degradation reasons. All of those reasons, from the point of view of safety, process, equipment, control, and product quality, dictate the rhythm in a plant environment. Hence, IPA’s and acetone’s ignition temperatures are 425 °C^64^ and 465 °C,^65^ respectively. Using Aspen Plus v.12.1 sensitivity analysis, the reactor’s operating temperature was varied from 330 to 430 °C in 0.5 °C steps.

### Biomass Usage + Reactor Temperature Optimization

The last studied scenario is the combination of biomass usage and reactor temperature variation. The goal is to reduce even more the operating costs and mitigate environmental impacts. The same modifications done in the last two sections were combined here.

## Methods

### Process Simulation and Considerations

In order to evaluate the different process scenarios considered in this work, all of them were simulated in Aspen Plus v.12.1. All of the components included in the simulation were already available in the software database. HPS, LPS, and reactor fuel in conventional routes (Fired Heat 1000—FH1000) were present in the database. Biomass was added and configured to be used as a utility.

Aiming to evaluate all process alternatives fairly, some assumptions were made. They are important to the mathematical representation of the process in the applied software. In every scenario and case, the same specifications of the original process were kept, except in the analyzed systems.

### Thermodynamic Package

Aspen Plus contains a series of thermodynamic package with specific characteristics. To evaluate the robustness and choose the most appropriate thermodynamic model, three combinations were tested due to nonidealities in the vapor phase. They were as follows:

- Universal Quasichemical (UNIQUAC) package with the Soave–Redlich–Kwong (SRK) equation of state for the vapor phase.^66^
- Non-random two-liquid (NRTL) package with the SRK equation of state for the vapor phase.^67^
- UNIQUAC package with the Peng–Robinson equation of state with Boston–Mathias modification for the vapor phase, due to its better applicability for mixtures and compounds above their critical temperature (IPA’s critical temperature is 235.15 °C).^68–70^

However, with the first two thermodynamic packages, we were not able to reach the acceptable acetone purity fraction, as indicated by ref 37. With the first one, the calculations did not converge, while the second resulted in only 95% acetone purity, even with the same parameters from the reference work.^37^

In order to obtain a literature-supported base-case model with which to compare our proposed modifications, we opted for choosing the thermodynamic package that returned the closest results. Therefore, we used UNIQUAC, which was the same thermodynamic package that was used in the reference,^37^ combined with the Peng–Robinson equation of state with Boston–Mathias modification for the vapor phase, achieving a 99.99% purity in the distilled acetone stream.

### Reaction Kinetics

The stoichiometry of IPA dehydrogenation resulting in acetone is shown in eq 1. The reaction kinetic model adopted is the same as in ref 37, with the same values for rate constant and activation energy, aiming to reproduce the results indicated by the reference. It is a first-order reaction model that follows the Arrhenius equation as shown in eq 2, where rIPA is the reaction rate; *k*_0_ is the pre-exponential constant (3.51 × 10^5^ m_gas_^3^/(m_reactor_^3^·s)); *E*_a_ is the activation energy (72.38 MJ/kmol); *R* is the ideal gas constant (8.314 × 10^–3^ MJ/(kmol·K)); *T* is the temperature, in kelvin; and CIPA is the molar concentration of IPA, in kmol/m_gas_^3^.

1 $${\left(CH_{3}\right)}_{2}CHOH\to {\left(CH_{3}\right)}_{2}CO+H_{2}$$

2 $$-r_{IPA}=k_{0}\times \exp\left[-\frac{E_{a}}{RT}\right]\times C_{IPA}$$

The referenced work comments that the reaction occurs in the gaseous phase on a nonspecified catalyst.^37^ After a literature search, CuSiO_2_ was assumed to be this catalyst due to its capacity to achieve high conversion rates on IPA hydrogenation, besides high selectivity, avoiding undesired side products. Its apparent density is 875 kg/m^3^.^71^

Typical reaction conditions are 2 bar and 350 °C, the conversion rate is between 85% and 92%, and the reaction duty is 62.9 kJ/mol.^37^ To achieve a high reaction yield, a hot stream is used to heat the reactor and keep the temperature in the typical condition range. In this work, the hot stream fuel source varied according to the route, as explained. If the conditions are not controlled and a nonselective catalyst is used, undesired side reactions may occur, e.g., IPA dehydration to propene.^72^

### Conventional Process Simulation

The equipment units depicted in Figure 1 were simulated according to the specifications presented in the Process Description section.^37^ A maximum deviation of 5.0% was tolerated in comparison with the temperature, pressure, flow rate, and molar fraction values reported in the reference, although higher deviations were observed for some streams and are depicted in Table S17 of the Supporting Information.

It is worth mentioning that there is a furnace providing a hot molten salt stream to heat the reactor.^37^ However, in the process simulations performed in this work, for the sake of simplicity, it was replaced with a heated reactor to simulate the same heating effect, with satisfactory results (inefficiencies were neglected). Aspen Plus does not have the functionality to choose a heating utility for the reactor in the simulation; therefore, the heating stream in the reactor was not simulated, but it was considered when calculating expenditure and operational costs, according to the used fuel source. The pieces of equipment P-1102A/B, P-1103A/B, P-1105A/B, V-1103, V-1104, E-1105, and E-1107 were disregarded from the simulation since the furnace was replaced, and the distillation models already have condensers on their top and reboilers at their bottom, representing the required heat exchangers, pumps, and reflux tanks. Also, V-1101 was disregarded from the simulation since it did not greatly influence the steam properties and process functionality. The resulting process flowsheet is depicted in Figure 2. The methodology to simulate all scenarios of this work is described in Table 1.

**Figure 2:**
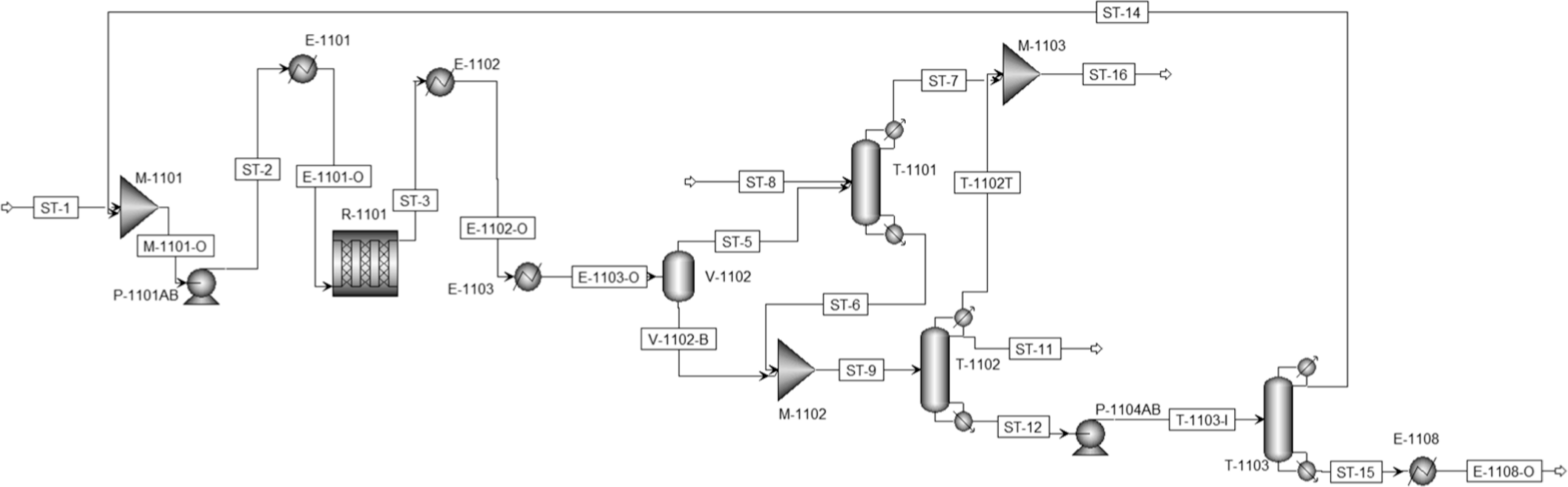
Conventional process flowsheet adapted for simulation in Aspen Plus.

**Table 1:** Methodology That Was Used to Simulate the Acetone Production

item	methodology
description	simulation of acetone production via the dehydrogenation of isopropyl alcohol
objective	to obtain quantitative data about the process to estimate CO_2_ emissions, utility consumption, and economic and sustainability indicators for further comparison between scenarios
reference	Turton^37^
simulation software	Aspen Plus v.12.1
thermodynamic package	UNIQUAC combined with the Peng–Robinson equation of state with Boston–Mathias modification
operation	stationary state
distillation columns	T-1102: number of stages = 66; overhead pressure = 1.20 bar; bottom pressure = 1.40 bar; feed stage = 33
	T-1103: number of stages = 19; overhead pressure = 1.20 bar; bottom pressure = 1.40 bar; feed stage = 16
components	isopropyl alcohol, water, hydrogen, and acetone
product	stream ST-11: acetone with a molar fraction of 0.999
validation criteria	a maximum deviation of 5.0% was tolerated in comparison with mass and energy streams from Turton^37^
considerations	vaporizer uses HPS or biomass, depending on the scenario
	reboilers use LPS or biomass, depending on the scenario
	condensers and coolers use CW
	chiller (E-1103) uses river water (CHILLED)
	reactor (R-1101) uses FH1000 or biomass, depending on the scenario
	electric pumps operate with an adiabatic efficiency of 40%
	heat exchangers do not have pressure drop
	all these considerations were the same as those used by the reference work of Turton^37^ and the references they followed^73,74^

### Biomass Usage Simulation

The same simulation was conducted in this route, however, by configuring and using biomass as a heating utility source, as explained in the Biomass Usage section. Mass and energy results are the same for the process streams; what will change are the subsequent economic and sustainability analyses.

A default biomass value must be set. Therefore, biomass’ composition is defined as the obtained mean values considering “all varieties of biomass”,^7^ hence: C: 51.3%, O: 41.0%, H: 6.3%, N: 1.2%, and S: 0.19%. This composition was used to calculate its HHV in eq 3, using the developed ultimate analysis correlation,^63^ where HHV is in MJ/kg, C is carbon dry weight percentage concentration, H is hydrogen dry weight percentage concentration, and O is oxygen dry weight percentage concentration. The reference considered N, S, and Cl dry weight percentage concentrations as part of O’s, and then they must be added to it.

3 HHV=−1.3675+0.3137×C+0.7009×H+0.0318×O

### Reactor Temperature Optimization Simulation

As stated before, the acetone production reaction is endothermic: heat must be supplied in order to shift the reaction equilibrium to the product side. Therefore, there is a heating jacket surrounding the reactor to provide the necessary heat. Aiming to analyze the influence of the reaction temperature on this process and if it is possible to change the temperature reported by the reference and obtain better results within safety parameters without compromising the product’s quality, a case study was conducted. The reactor’s temperature was varied, hence the fuel streamflow rate that is responsible for the reaction duty. Its influence on the process was analyzed by observing the reactor’s acetone outlet versus the reaction’s temperature and, consequently, the fuel cost.

### Biomass Usage + Reactor Temperature Optimization Simulation

The last scenario was simulated by combining the biomass usage and the reactor temperature optimization (TOpt) ones. The same modifications described in the last two sections were combined here.

### PI System

If those simulations were a real and live plant process from a company, those data would be available in real-time at a controller program and probably online at a data historian, hence, PI System. However, it only makes sense to use the PI System in a dynamic environment. As all simulations were conducted in the steady state, one extra simulation of the reaction section was made in Aspen Dynamics v.12.1. The same inlet conditions, reaction, and reactor specifications were considered. The reactor’s temperature was varied from 350 to 450 °C. The control parameters were not the subject of deep study in this project and were defined to have a slow response, since the residence time in the reactor is small (10 s); hence, gain = 1%/%, integral time = 10 min, derivative time = 10 min, and controller action was defined as “reverse”. The reactor was initialized at 350 °C, and at some point, its set point was changed to 450 °C. The system started executing, and after it achieved the set point and stabilized, its set point was set again to 350 °C. The process was conducted until it stabilized again at the new set point. This was done to simulate a process failure, where the heat stream temperature was above the limit, and an action was taken to correct it, bringing it back to regular operating conditions and stabilizing it, keeping it within the safe operating limits.

A hierarchy was modeled on the PI Asset Framework to represent the reactor and its main variables, e.g., inlet and outlet stream molar flow rates, inlet and outlet pressures and temperatures, reaction rate, and reactor duty. They were built in the form of a template to allow replicability in case there were any other pieces of equipment on site or similar facilities. Those variables were mapped as PI Points (tags) to the respective attributes in the element. The dynamic simulation provided data for some of them, while others were static (informative) data, stemming from the reference work.^37^ The remaining data that were not provided, such as fuel mass flow, fuel cost rate, heat loss, and CO_2_ emission rate, were calculated via PI Asset Framework analysis, a feature of PI System, which allows using system variables in more complex calculations and generating new calculations that did not exist before, depending on the process understanding and monitoring. Also, Event Frames, which are alerts, were generated to keep plant personnel aware of process deviations, such as temperatures above high operation limits. In that alert, the same reactor’s main variables and other auxiliary ones were captured to help plant personnel identify process bottlenecks and deepen their process analysis. In addition to this alert, the operator would also receive an e-mail with an overview of the process anomaly and an external link to a closer look at it.

On top of it, to simulate how an operator would deal with it on a daily basis, a PI Vision dashboard was developed, containing useful variables. This would enable the operator to have the dashboard on a computer and monitor the process over time. Also, it would be possible to plot variables’ trends against each other and analyze their behavior and consequences across time.

### Economic Analysis

Equipment sizing was performed from the results of the mass and energy balances of the process simulations. Each piece of equipment’s dimensions and main properties are depicted in Tables S3–S12 of the Supporting Information. Those properties were either specified in the reference or calculated with supplementary formulas and approximations. It should be emphasized that the present work is a feasibility study whose goal is to compare process scenarios and analyze costs preliminarily; hence, the most accurate values could only be obtained in final project phases.

All equipment sizes and costs were determined with standard methodologies available in the process systems engineering literature.^2^ For column T-1101 (packed), the Leva method^2^ was applied for column diameter calculation, and HETP (height equivalent to a theoretical plate) methodology was applied to calculate the column height. However, for columns T-1102 and T-1103 (tray), the fair correlation was used to calculate the capacity parameter and the columns’ diameter. To determine their heights, the plate spacing is multiplied by the number of theoretical stages and divided by plate efficiency, which was considered constant along the column.

To calculate the vessels’ sizes, the methodology presented by Towler and Sinnott^75^ was implemented. That was the case with V-1102, while V-1103’s and V-1104’s costs were already included in the cost calculation of their respective columns.

The economic evaluation developed in this work considered the deduction of the capital investment necessary for acquiring all pieces of equipment involved in the plant; the deduction of the operational/production costs related to the plant; revenue estimation; annual cash flow; and product breakeven price, considering other market scenarios and comparing the different routes and scenarios.

All production costs were estimated based on an operation factor of 7920 h/year.^76^ December 2023 was used as the economic context for this work. Therefore, the prices of IPA and acetone were defined by it. Water and cooling water prices were considered to be the same. HPS, LPS, and FH1000 were defined by the simulator utilities’ database. Biomass, IPA, and acetone prices were obtained from Business Analytics IQ and Statista Web sites.^77–79^ For biomass (Statista^79^ considered “sawmill residue”, the most expensive one and therefore a conservative approach on this work’s side), it was assumed the average price of October 2023 (latest update), while acetone and IPA prices were from December 2023. These prices can be seen in Table 2. Also, if there was a positive cash flow in the process, the gross profit would have been discounted by the tax rate of 21%, a yearly average of the United States,^80^ to calculate the net profit.

**Table 2:** Prices of the Components Involved in the Processes

raw material/product/utility	unit	value	reference
HPS	US$/GJ	2.50	Aspen Plus v.12.1 Database
LPS	US$/GJ	1.90	Aspen Plus v.12.1 Database
biomass	US$/kg	0.03971	Statista^79^
FH1000	US$/GJ	4.25	Aspen Plus v.12.1 Database
river water	US$/GJ	5.00	Seider^2^
cooling water	US$/m^3^	0.027	Seider^2^
water	US$/m^3^	0.027	Seider^2^
IPA	US$/kg	0.755	IMARC Group^81^
acetone	US$/kg	1.467	IMARC Group^82^

### Capital and Production Costs

To estimate the total investment for the construction of the acetone production plant, the Lang method^2^ was used. In this method, the FOB (free on board) prices of each equipment unit are calculated and updated with the chemical engineering plant cost index.^83^ Then, the obtained total FOB price is multiplied by 1.05 to account for delivery of the equipment to the plant site. Lastly, this result is multiplied by the Lang factor, and thus, the fixed investment cost is obtained. These steps are summarized in eq 4, where *I*_F_ is the fixed investment; *f*_L_ is the Lang factor; PCI is the plant cost index for the project year; PCI_b_ is the plant cost index for the base year; and *C*_*i*_ is the FOB acquisition cost of equipment *i*.

4 $$I_{F}=1.05\times f_{L}\times \sum_{i}\left(\frac{PCI}{{PCI}_{b}}\right)C_{i}$$

The Lang factor in this case is equal to 5.04 for a plant that operates with fluids and does not include the working capital,^2^ and it was used so that the costs of installation, instrumentation and control, plumbing, electrical network, auxiliary constructions, land acquisition, construction and engineering costs, and involved fees were all considered. The working capital, which was established as 15% of the total investment, is deducted only in the first year of operation.

The equipment’s empirical FOB price estimation formulas, which are based on real industrial data, were obtained from Seider.^2^ They were made with data from 2013, for which the PCI_b_ was 567. Therefore, it was updated to December 2023, for which PCI was 789.6.

To estimate the production costs, the methodology of Seider^2^ was used. To obtain feedstock and utilities costs, one must multiply their mass or energy flow rates by their unit costs, presented in Table 2. Then, we multiply this result by the operation factor to obtain the annual values. Having the number of employees required per shift allows one to estimate the clerical labor cost by multiplying it by the hourly wage of the workers. The number of employees required per shift is calculated by eq 5, where *N*_wk_ is the number of workers per shift, *N*_s_ is the number of equipment that operate with solids—hence, 0 in this work—and *N*_ns_ is the number of equipment that do not involve particulate solids handling. It was the same across all of the simulation scenario.

5 $$N_{wk}={\left(6.29+31.7{N_{s}}^{2}+0.23N_{ns}\right)}^{0,5}$$

The production costs can be divided into three different categories: direct costs, indirect costs, and general expenses. Direct costs include raw materials, utilities, catalysts, supervisory and clerical labor, operating labor, maintenance and repairs, operational supplies, laboratory charges, and payment for the exploitation of patents and royalties. Indirect costs include overhead, packaging, storage, local taxes, and insurance costs. General expenses include administrative, distribution, and selling costs and research and development costs.

### Economic Evaluation Criteria

The economic evaluation criteria used in this work were fixed capital investment, total production costs (TPC), annual net profit, breakeven price of acetone, and potential savings with alert and process correction to malfunctioning/out-of-range operation using a PI System basic lease due. They allow one to understand the economic feasibility and attractiveness of each scenario. The first two criteria were described in the previous section.

To obtain the total revenue, one must multiply the amount of purified acetone, the only product of this plant, since hydrogen is stored only, by its market price and the annual operating hours. Then, one must obtain the gross profit by subtracting it from the total production cost, also calculated for one year of operation. At last, one multiplies the revenues by the income tax and discounts the gross profit by this value, resulting in the net profit. This determines whether the process is profitable (positive) or not (negative).

However, what confirms if the process is viable and helps determine if investments are returned by the profits is the net present value (NPV) being positive after the established project horizon, which was 20 years of operation, after 3 years of construction of the plant, investing 30%, 40%, and 30% of the fixed investment per year in the first years. In the fourth year, total production costs, revenues, and 15% of fixed investment (working capital) are considered. For the rest of the operation period, only total production costs and revenues are considered.

To obtain the break-even price of acetone for each run, its price was varied until NPV was equal to zero. The interest rate was set to 10%, an average of 2023’s USA fixed-income funds rate.^84^

Due to the reactor temperature optimization scenarios, apart from R-1101, some pieces of equipment were susceptible to minor cost deviations, hence heat exchangers and respective distillation columns. The rest were not influenced.

### Economic Sensitivity Analysis

Aiming to have a deeper understanding of how the raw materials, product, and main utilities prices influence the accumulated NPV, some price fluctuations (case studies) were individually conducted for each scenario. Those fluctuations were the variation of the prices of capital expenditure (CAPEX), IPA, acetone, natural gas, and biomass up and down by a factor of 10%. Also, to compare the difference between natural gas and biomass as fuel sources, their prices were compared, and the biomass’ price was varied up and down by a factor of 50%. One last analysis that was made was the evaluation of extreme biomass HHV. Based on the available data present in the biomass database,^7^ the ones with maximum and minimum HHV were evaluated as fuel sources as well and compared to the biomass and TOpt biomass scenarios. All these case studies of each scenario allowed the study of their influence on CAPEX, operating expenditure (OPEX), and NPV, consequently.

### Sustainability Analysis

In addition to the economic evaluation, a sustainability analysis was executed to provide a deeper report on the process characteristics. For sustainability analysis, a multicriteria methodology was implemented based on the green design criteria (GDC), which are qualitative, and on the scoring quantitative metrics (SQMs), which are quantitative.^85^

All criteria received the same weight ([Disp-formula eq1]). According to the process results, their adherence to a certain criterion would be classified as

- 1: High.
- 3: Medium.
- 9: Low/inexistent.

For SQM, a mean value for each variable was calculated based on the different scenarios. Then, each result must be divided by the normalized value (NV), which is given by dividing the respective value by the mean. According to the process results, their adherence to a certain criterion is calculated and classified as

- 1: NV < 1 high.
- 3: 1 ≤ NV < 3 medium.
- 9: NV ≥ 3 low/inexistent.

SQMs are explained in Table 3.

**Table 3:** SQMs’ Explanation

SQM metric	abbreviation	evaluation method
*E*-factor	EF	reactant mass flow rate divided by the product mass flow rate minus 1
carbon intensity	CI	produced CO_2_ mass flow rate divided by the product mass flow rate
thermal intensity	TI	sum of all heating power divided by the product mass flow rate (do not consider cooling energy streams)
water intensity	WI	water mass flow rate divided by product mass flow rate

On the WI metric, besides the water used in ST-8, the cooling and heating water (or vapor) losses were also considered. Water losses were assumed as 1% of the cooling utilities mass flow rates and 10% of the heating utilities mass flow rates.^2,37^

GDC metrics are explained in Table 4.

**Table 4:** GDC Metrics’ Explanation

GDC metric	evaluation method
GDC01	preventing rather than treating waste is the best environmental protection strategy
GDC02	incorporating all materials used in the process into the final product, designing tailored to needs with maximum efficiency
GDC03	using and generating substances that possess little or no toxicity to human health and the environment while preserving efficacy of use
GDC04	recognizing environmental and economic impacts of energy requirements and minimizing them
GDC05	using renewable rather than depleting materials
GDC06	avoiding unnecessary derivatization and minimizing complexity in products
GDC07	avoiding byproducts by using catalytic reactants as selective as possible
GDC08	product should not persist in the environment after usefulness. Targeted durability, not immortality, should be a design goal
GDC09	real-time, in-process monitoring and control to minimize pollution and release of hazardous substances
GDC10	embodying a holistic, systems approach to risk reduction
GDC11	complexity must be viewed as an investment when making design choices on recycle, reuse, or beneficial disposition
GDC12	material diversity in multicomponent products should be minimized to promote disassembly and value retention
GDC13	designing for performance in a commercial “afterlife” and designing for disassembly
GDC14	holistically designing with innovative solutions while meeting geographic and cultural specificities

Based on US-EPA-Rule-E9-5711, the CO_2_ emission calculation was standardized, considering 93.80 kg of CO_2_/million BTU for biomass and 53.60 kg of CO_2_/million BTU for HPS, LPS, and FH1000 (source: natural gas) and an efficiency factor of 85% for all heating utilities.^86,87^

Possessing the grades for these two methodologies, we require some additional steps to calculate the sustainability degree (SD) of every process. The mean of all GDC grades, with respect to the respective process, is calculated (criteria that are not encompassed by the studied process received 0 and are not considered in the mean). Then, a multicriteria severity matrix (SM) is calculated, multiplying those means by each grade from SQM, in respect to the process they belong to. Next, a criticality factor (CF) matrix is calculated by adding up all the values per row from SM. To calculate the severity index (SI) vector, first, one must choose the process to provide the reference criticality factor (cref). The conventional route was chosen in this work. Afterward, every value from the CF matrix is multiplied by 100 and divided by cref. These values indicate the severity percentage of the process alternatives in comparison to the reference one. Last, to calculate the SD matrix, one calculates the inverse of all of the values from the SI matrix and multiplies them by 100. SD indicates how many times a process is more (if greater than one) or less (if smaller than one) sustainable than the reference process. These steps are further explained in the work of de Queiroz Fernandes Araújo.^85^

## Results and Discussion

### Reactor Temperature Optimization Results

The reference work claims that it is possible to separate 99.9% pure acetone in the distillate of T-1102, with a reflux ratio (RR) of 2.245, a distillate-to-feed ratio (D/F) of 0.436, and a molar flow rate of 32.29 kmol/h.^37^ However, it was not possible to achieve those values in the simulation. Even if we could reach one of those ratios, the other one would not follow up, and the purity of acetone would drop to around 85%. Therefore, 99.9% pure acetone was obtained with an RR of 0.600, a D/F of 0.350, and a molar flow rate of 27.36 kmol/h. This setup was the same for the biomass usage scenario. It is important to emphasize that there is no experimental data available in order to compare the obtained results with, apart from the reference work.^37^ Also, there are no real process data to which the obtained results could be compared.

However, for the reactor temperature optimization (TOpt) scenarios, the optimum calculated temperature was 375.5 °C, producing more acetone. Consequently, D/F must be higher to separate it, being equal to 0.370, which increases the molar flow rate to 28.78 kmol/h.

The comparison between the reactor outlet acetone mole flow rate (ST-3) and the 99.99% acetone mole flow rate on the distillate stream (ST-11) from T-1102, with D/F equal to 0.350, as a function of the reactor’s temperature is shown in Figure 3. Figure 4 also compares ST-3 and ST-11, but with T-1102’s D/F equal to 0.370. It is possible to notice that as the acetone production reaction is an endothermic reaction, a higher reaction temperature allows a higher production of acetone.

**Figure 3:**
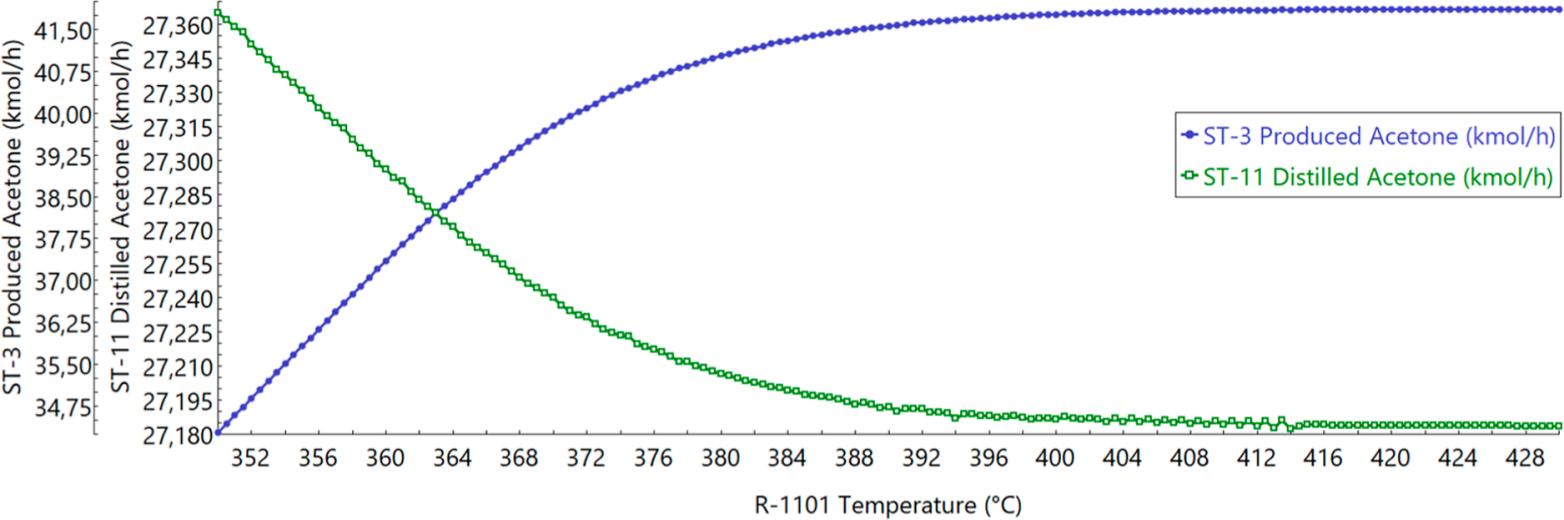
Produced and distilled acetone molar flow rates depending on temperature with a D/F of 0.350.

**Figure 4:**
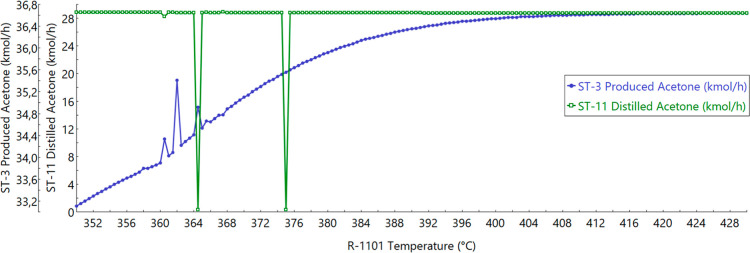
Produced and distilled acetone molar flow rates depending on temperature with a D/F of 0.370.

As noted in Figure 3, even though more acetone is produced, T-1102 is not able to separate it properly, decreasing its distillate molar flow rate. Although Figure 3 shows a peak of acetone production at 410 °C, and then it stabilizes due to reactant quantity limitation, T-1102 was not able to separate more acetone, even by manipulating its RR, D/F, and size. If the reactor temperature were raised to the peak, it would consequently be a waste of utility and acetone, since it would not be able to enhance the distillate stream with the desired purity. In order to separate the acetone more in T-1102, a higher D/F was needed. Therefore, the D/F for the TOpt scenarios was 0.370, increasing the distillate flow, as depicted in Figure 4. In Figure 4, acetone production also peaks at 410 °C. Its behavior prior to 375.5 °C is interpreted as error by the simulator and should not be accounted for in the analysis.

### Economic Results

All scenarios follow similar economic behavior, concentrating most of the equipment cost on towers and the reactor. The reactors in the TOpt scenarios are more expensive than the ones in the conventional and biomass scenarios. This increases their values by 2% in comparison to those of the baseline. Some minor alterations are noticed on towers, pumps, and HEXs as well, as depicted in the equipment cost percentage distributions in Figure S1 of the Supporting Information and on the equipment cost overview in Table 5. On the matter of fixed investment, as equipment sizing is the major influencing factor on it, and since the TOpt scenarios have a reactor that exchanges more heat, their fixed investment is slightly higher, 1.5%, than the conventional one, which is the baseline.

**Table 5:** Comparison between the Equipment Costs from Different Scenarios

equipment	unit	conventional	biomass	TOpt conventional	TOpt biomass
P-1101	US$	17,841.70	17,841.70	17,839.84	17,839.84
P-1104	US$	18,435.14	18,435.14	18,423.04	18,423.04
T-1101	US$	62,536.97	62,536.97	62,663.33	62,663.33
T-1102	US$	183,929.73	183,929.73	184,767.54	184,767.55
T-1103	US$	109,137.68	109,137.68	108,180.68	108,181.44
V-1102	US$	9,453.38	9,453.38	9,342.49	9,342.51
R-1101	US$	211,722.70	211,722.70	227,185.15	227,185.37
E-1101	US$	28,545.15	28,545.15	28,545.15	28,545.15
E-1102	US$	28,620.32	28,620.32	28,590.08	28,590.08
E-1103	US$	30,096.78	30,096.78	30,349.39	30,349.43
E-1108	US$	3,438.29	3,438.29	3,389.20	3,389.20

All scenarios that use biomass as a heating utility fuel source presented lower utilities costs, at least 25.00% lower than the baseline, because biomass is a cheaper fuel source. The comparison between the utilities’ hourly costs from conventional and biomass scenarios is depicted in Figure 5. However, reducing utilities did not impact TPC much, as depicted in Table 6.

**Figure 5:**
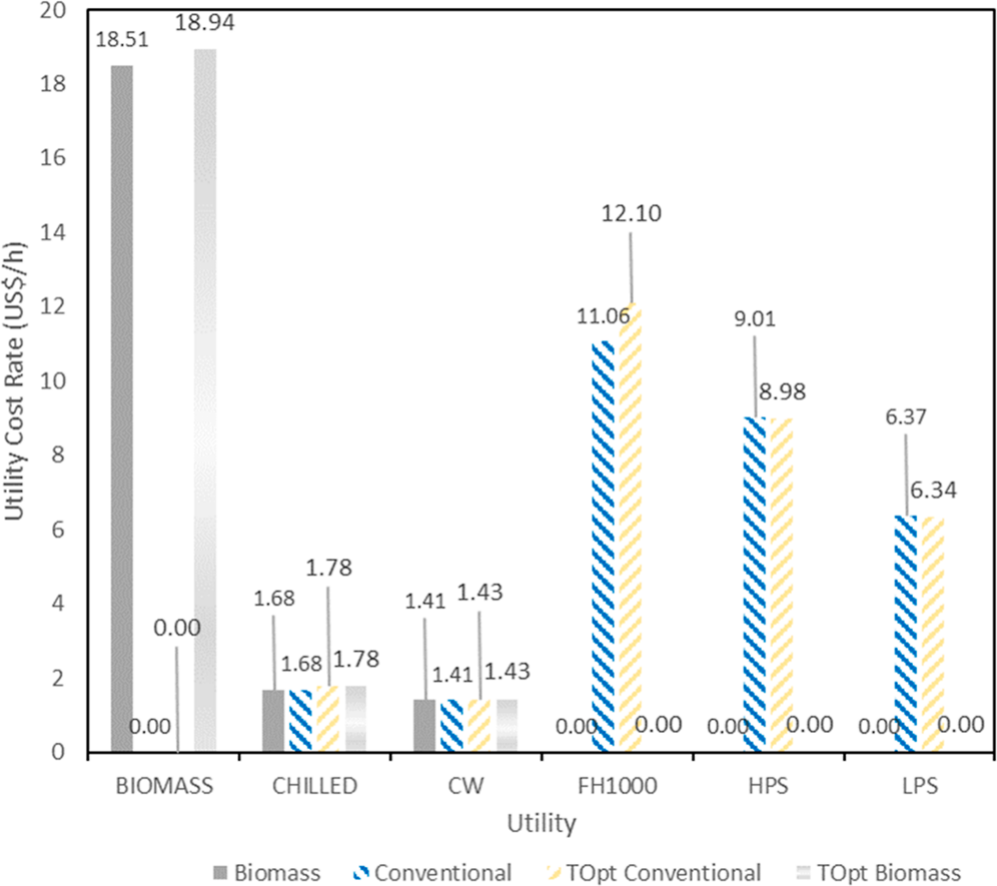
Comparison of hourly utility cost from conventional and biomass scenarios.

**Table 6:** Comparison between Economic Indicators and Their Deviations from All Scenarios

source	unit	conventional	biomass	TOpt conventional	TOpt biomass
fixed investment	US$	3,724,286.48	3,724,286.48	3,806,407.98	3,806,413.54
fixed investment deviation	%			2.00	2.00
total production cost	US$	18,180,579.58	18,103,987.39	18,214,638.65	18,132,735.84
total production cost deviation	%		–0.42	0.19	–0.26
utilities cost	US$	233,877.60	171,072.00	242,589.60	175,428.00
utilities cost deviation	%		–27.00	4.00	–25.00
revenues	US$	18.46 × 10^6^	18.46 × 10^6^	19.42 × 10^6^	19.42 × 10^6^
revenues deviation	%		0.0	5.2	5.2
cash flow	US$	223,188.58	283,696.42	949,288.99	1,013,992.20
cash flow deviation	US$		27	325	354
NPV	US$	–1.70 × 10^6^	–1.21 × 10^6^	4.10 × 10^6^	4.63 × 10^6^
NPV deviation	%		29	341	372
acetone breakeven price	US$/kg	1.49	1.48	1.42	1.41
acetone breakeven price deviation	%		–1.00	–4.69	–5.00

For all scenarios, the raw material costs are the same, hence US$12,511,462.23/year. It is important to emphasize that they, mainly due to IPA, are responsible for at least 66.0% of TPC. Although all scenarios are profitable, the yearly positive cash flows from conventional and biomass scenarios are not sufficient to compensate for the investments during the project horizon, even though the last one spends less TPC than the baseline (0.42%).

TOpt conventional and TOpt biomass scenarios spend 0.19% more and 0.26% less TPC than the baseline, respectively. However, they have greater revenues (5.2% more), increasing their annual cash flows to around US$1 M, which compensates for the investment during the project horizon. Their NPVs are at least 341 times higher than the baseline, proving themselves as great opportunities to improve the process within the economic scenario. The most profitable scenario is TOpt biomass, since it spends 0.26% less TPC than the baseline, achieving the highest NPV among all scenarios: US$4.63 M.

To absolutely consider an investment advantageous and profitable, its NPV must be generally greater than or equal to zero in a period of five operating years (after the three construction years, totaling eight years) at most. Conventional, biomass, and TOpt conventional scenarios do not fall under this case and would need to sell acetone at least at US$1.54/kg, US$1.54/kg, and US$1.47/kg, respectively, to achieve it. In the fifth year of operation, only TOpt biomass was able to compensate all investments, achieving a NPV of US$71,029.89, confirming its first place in attractiveness due to its higher profitability, whereas TOpt conventional would only reach a positive NPV (US$347,378.98) in the sixth year of operation. Nevertheless, both TOpt alternative scenarios are attractive and feasible and make the process profitable in less time than the project horizon.

### Economic Sensitivity Results

After varying the prices of IPA, acetone, CAPEX, natural gas, and biomass, it is possible to notice the influence of those variables in the accumulated NPV after the established project horizon. Increasing CAPEX- and OPEX-related costs and decreasing revenues prices have the tendency to decrease NPV, while decreasing CAPEX- and OPEX-related costs and increasing revenues prices have the tendency to increase NPV. However, this increment may not be able to make the process economically viable. Those variations directly influence the scenarios’ economic viability, and their outcome will be discussed further.

In the conventional and biomass scenarios, the case studies that increased the acetone selling price and reduced IPA cost were the ones that achieved NPV greater than zero. Although increasing/decreasing CAPEX cost and increasing/decreasing natural gas/biomass cost case studies were profitable (positive annual cash flow), they did not achieve NPV greater than zero. Increased IPA price and decreased acetone price case studies were not profitable and did not achieve NPV greater than zero, consequently.

In the TOpt conventional and TOpt biomass scenarios, the case studies that increased the acetone selling price, reduced IPA cost, increased/decreased CAPEX cost, and increased/decreased natural gas/biomass cost were the ones that achieved NPV greater than zero. Increased IPA price and decreased acetone price case studies were not profitable and did not achieve NPV greater than zero, consequently.

On the matter of utility prices, the case studies comparing biomass and natural gas prices showed how this hot utility influences the total utilities cost. Considering the average biomass price in the USA (39.71 US$/metric ton) and dividing (and properly converting) it by the average HHV (20.49 MJ/kg), it resulted in 0.001938 US$/MJ (or 1.938 US$/GJ), while the LPS cost is 1.9 US$/GJ and the HPS cost is 2.5 US$/GJ, respectively, both met with natural gas in the base scenario. As the biomass price is close to the LPS cost and around 75% of the HPS cost, the utilities’ cost falls considerably, since hot utility streams are the most expensive ones in this work.

To evaluate the economic feasibility of the process even in cases of fluctuations in biomass prices, we varied the biomass price up and down by a factor of 50%. In the biomass scenario, decreasing biomass’ price by 50% also decreased utilities cost (by 43%) and increased accumulated NPV (by 47%) and cash flow (by 25%). In the same scenario, increasing biomass’ price by 50% resulted in the complete opposite results. Those results were compared to the base biomass scenario. None of these case studies achieved NPV >0. More costs need to be reduced or more revenues must be generated to reach a positive NPV. However, in the TOpt biomass scenario, decreasing biomass’ price by 50% also decreased utilities cost (by 43%) and increased accumulated NPV (by 13%) and cash flow (by 7%). In the same scenario, increasing biomass’ price by 50% resulted in the complete opposite results. Those results were compared to the base TOpt biomass scenario. All these TOpt biomass case studies achieved NPV >0. This scenario is economically viable even in situations of intense utility price fluctuation.

Regarding biomass composition, as was said before, the “mean” one was used, resulting in a HHV of 20.44 MJ/kg. Considering the extreme samples from the biomass available database,^7^ we calculated the lowest and the highest HHV: they are 16.70 MJ/kg for pepper residue and 29.24 MJ/kg for greenhouse-plastic waste, respectively. Finding CO_2_ emission factors and prices for these other two sources was not possible, and we used CO_2_ emission factors considering “biomass-derived fuels (solid)”, according to “Environmental Protection Agency—Mandatory Reporting of Greenhouse Gases”^86^ (that is the same one used for the “mean” biomass).

As their HHVs are different, they have different impacts on the utilities cost. Considering the biomass scenario, while the “mean” biomass spends around US$171 k in utilities, the pepper residue one spends around US$204 k, and the greenhouse-plastic waste one spends US$127 k. In the TOpt biomass scenario, while the “mean” biomass costs around US$175 k in utilities, the pepper residue one spends around US$209 k, and the greenhouse-plastic waste one spends US$130 k. Therefore, using a biomass source with a higher HHV may contribute to spending less of the OPEX. None of the new Biomass case studies achieved NPV >0, though. Both TOpt biomass case studies achieved NPV >0. However, more accurate data is needed to precisely calculate the CO_2_ emissions.

The comparison between the accumulated NPV (after the project horizon) from each new case study, the payback time (in years), and the economic viability of each one are depicted in Table 7.

**Table 7:** Comparative Analysis of NPV for Several Price Fluctuation Case Studies

case study	accumulated NPV (10^6^ US$)	payback (y)	economically viable
biomass	–1.21		false
acetone decreased 10%	–13.01		false
acetone increased 10%	10.60	6	true
CAPEX decreased 10%	–0.18		false
CAPEX increased 10%	–2.24		false
biomass decreased 10%	–1.10		false
biomass decreased 50%	–0.64		false
IPA decreased 10%	8.55	6	true
IPA increased 10%	–10.97		false
maximum HHV	–0.87		false
minimum HHV	–1.47		false
biomass increased 10%	–1.32		false
biomass increased 50%	–1.78		false
conventional	–1.70		false
acetone decreased 10%	–13.51		false
acetone increased 10%	10.11	6	true
CAPEX decreased 10%	–0.67		false
CAPEX increased 10%	–2.73		false
IPA decreased 10%	8.06	7	true
IPA increased 10%	–11.46		false
natural gas decreased 10%	–1.54		false
natural gas increased 10%	–1.86		false
TOpt biomass	4.63	8	true
acetone decreased 10%	–7,79		false
acetone increased 10%	17.04	5	true
CAPEX decreased 10%	5.68	8	true
CAPEX increased 10%	3.57	10	true
biomass decreased 10%	4.74	8	true
biomass decreased 50%	5.21	8	true
IPA decreased 10%	14.38	5	true
IPA increased 10%	–5.13		false
maximum HHV	4.98	8	true
minimum HHV	4.36	9	true
biomass increased 10%	4.51	8	true
biomass increased 50%	4.04	9	true
TOpt conventional	4.10	9	true
acetone decreased 10%	–8.32		false
acetone increased 10%	16.52	5	true
CAPEX decreased 10%	5.15	8	true
CAPEX increased 10%	3.05	10	true
IPA decreased 10%	13.86	6	true
IPA increased 10%	–5.66		false
natural gas decreased 10%	4.27	9	true
natural gas increased 10%	3.93	9	true

### Sustainability Results

Due to using biomass and varying the reactor’s fuel usage, every scenario has some particularities regarding the sustainability indicators. The grades of the scoring quantitative metrics are compared in Table 8. Green design criteria’s results are stated in Table 9.

**Table 8:** Comparison of All Scenarios’ SQM Grades

metric	conventional	biomass	TOpt conventional	TOpt biomass
CI grade	3	1	3	1
EF grade	3	3	1	1
TI grade	3	3	1	1
WI grade	3	1	3	1

**Table 9:** Comparison of All Scenarios’ GDC Grades

criteria	conventional grade	biomass grade	TOpt conventional grade	TOpt biomass grade
criterion 1	1	1	1	1
criterion 2	1	1	1	1
criterion 3	1	1	1	1
criterion 4	3	1	9	1
criterion 5	9	1	9	1
criterion 6	1	1	1	1
criterion 7	1	1	1	1
criterion 8	1	1	1	1
criterion 9	0	0	0	0
criterion 10	0	0	0	0
criterion 11	1	1	1	1
criterion 12	1	1	1	1
criterion 13	0	0	0	0
criterion 14	0	0	0	0
total	20	10	26	10

On the SQM matter, both TOpt scenarios had better results for EF and TI than conventional and biomass scenarios because they produced more acetone, which lowered those numbers.

Biomass and TOpt biomass scenarios had better grades for WI due to the greater higher heating value biomass has in comparison to FH1000, having fewer losses in heating processes. Although biomass and TOpt biomass scenarios would have emitted 1259 kg CO_2_ and 1287 kg/h CO_2_, respectively, considering carbon neutrality, which states that a source of carbon emissions, hence (burning) biomass, consumes the same amount of CO_2_ when growing as it emits when completely burnt,^9^ it can be considered that it does not contribute to the carbon intensity calculation, emitting zero CO_2_. Therefore, it provided a high adherent grade for them, while conventional and TOpt conventional got worse grades, as the energy heat source (natural gas) emitted non-net zero CO_2_.

On the GDC side, almost all criteria had the same grade for all scenarios, apart from criteria 4 and 5, which dictated the difference between their final sums. The difference in their grades is noted in Table 9. It is important to emphasize that not all GDCs are encompassed by this process; therefore, those criteria received a grade of zero.

All criteria, besides 4 and 5, received the same grade because they refer to aspects that do not change between the scenarios. According to their definition in the Sustainability Analysis section, the reasons behind their grades are explained in Table 10.

**Table 10:** Justifications for the Grades Are Attributed to GDC Metrics

GDC metric	justification
GDC01	wastewater containing traces of IPA and acetone; needs to be treated before being discarded but has low toxicity^88,89^
GDC02	grade based on the conversion ratio: reactor raw material feed/reactor product outlet; higher than 95% to be considered as high adherence
GDC03	raw material: IPA; product: acetone; low toxicity^88,89^
GDC04	varies according to the scenario
GDC05	varies according to the scenario
GDC06	simple and direct synthesis scenario
GDC07	reaction-specific catalyst used
GDC08	acetone is volatile at environmental pressure and temperature
GDC09	not considered in this work
GDC10	not considered in this work
GDC11	recycle to use remaining reactant
GDC12	only one product and one byproduct; efficient separation from other components
GDC13	not considered in this work
GDC14	not considered in this work

Criteria 4 and 5 are dependent on the process and are explained by the fuel source. As more fuel is being used and it is a fossil fuel (natural gas), TOpt conventional received a poor adherence grade on criterion 4. On the other hand, both biomass scenarios received a high adherence grade because they minimize the environmental impacts. Regarding criterion 5, biomass scenarios received a high adherence grade due to the use of biofuel. The biomass scenarios scored the lowest summation of values, 50% less than the baseline, meaning that they damage the environment less, while TOpt conventional scored the highest one, 30% more than the baseline, damaging the environment more.

It can be concluded that, on the quantitative matter, the TOpt scenarios were the best options because they had better indicators, produced more acetone, and achieved higher adherent grades. However, on the qualitative ones, both biomass scenarios were equal, and TOpt conventional was the worst for the environment.

Possessing these quantitative and qualitative grades, CF, SI, and SD, the sustainability degrees, are calculated. They are shown in eqs 6, 7, and 8, respectively.

6 $$CF=\left[\begin{array}{l} \begin{array}{l} \begin{array}{l} 24\\ 8 \end{array}\\ 20.8 \end{array}\\ 4 \end{array}\right]$$

7 $$SI=\left[\begin{array}{l} \begin{array}{l} \begin{array}{l} 100\\ 33.34 \end{array}\\ 86.67 \end{array}\\ 16.67 \end{array}\right]$$

8 $$D=\left[\begin{array}{l} \begin{array}{l} \begin{array}{l} 1\\ 3 \end{array}\\ 1.15 \end{array}\\ 6 \end{array}\right]$$

Each row corresponds to a process, and the order is conventional, biomass, TOpt conventional, and TOpt biomass. According to SD results, it is noted that all alternative process scenarios are more sustainable than the baseline, which is the conventional one. Even the TOpt conventional, which was allegedly more harmful to the environment than it (according to the GDC), showed itself more sustainable when the metrics were combined. Nevertheless, both scenarios that use biomass are more sustainable than the others, with TOpt biomass being the most sustainable one, by a factor of 6, in comparison to the baseline. This scenario excelled in all grades and criteria, being more environmentally attractive, since it produces more acetone while minimizing the impact on the environment.

### PI System Results

As was demonstrated, turning this process economically feasible required some analysis and optimization. The simulator made it possible, but there are some parameters that would have been easier to calculate and identify with a data historian, especially if the data came from a real and live plant, allowing the comparison between the different scenarios and case studies through analyses (calculations). Therefore, the PI System Asset Framework structure supported the construction of a PI Vision dashboard, as depicted in Figure 6a, that would be overseen by an operator daily. This dashboard contains the most important pieces of information about their area of work; hence, the reaction section overview. It shows a 4.5 h overview of the process in which an anomaly was simulated and corrected, as described in the PI System section, and will be described and explained further.

**Figure 6:**
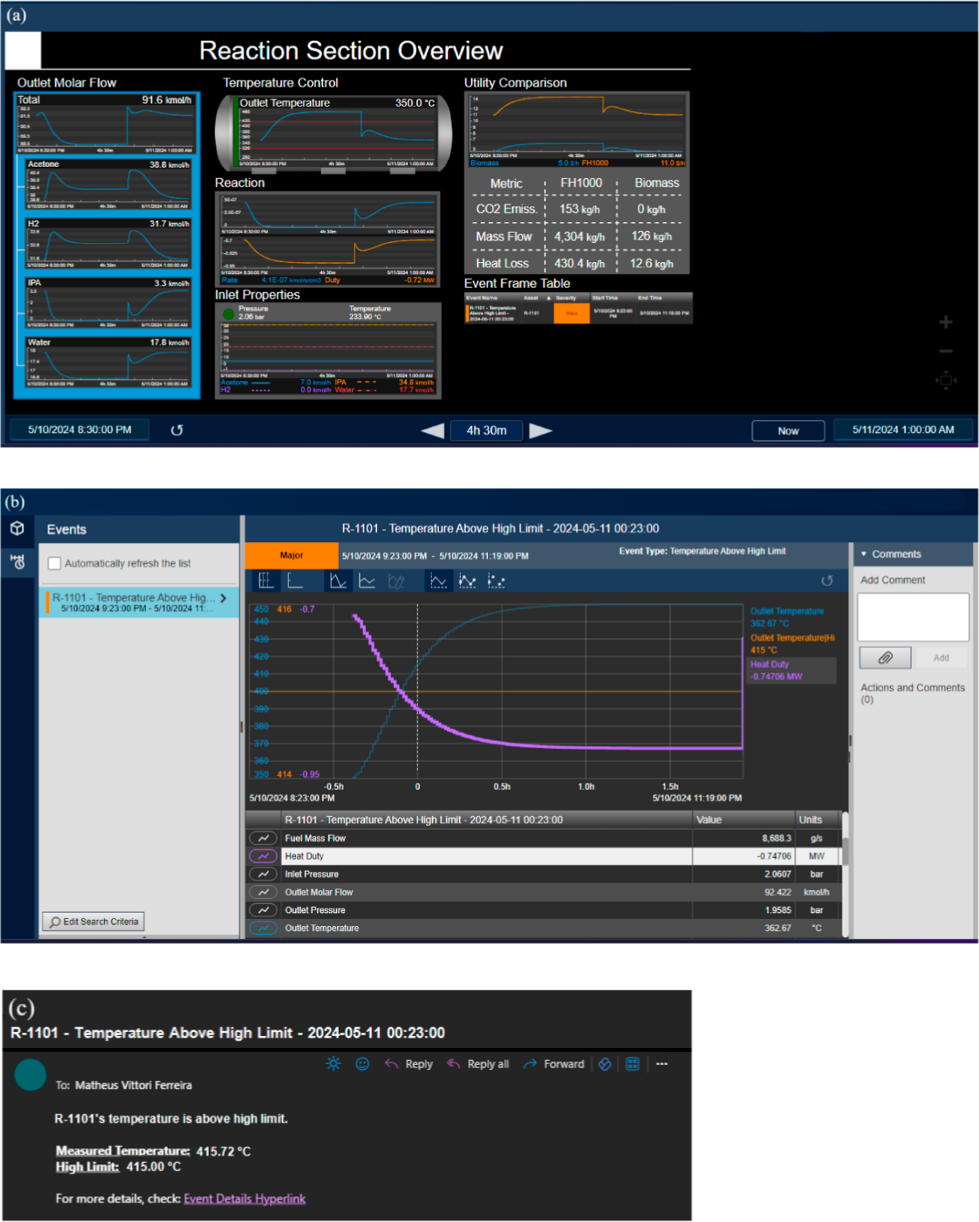
PI System features: (a) PI Vision dashboard of reaction section overview; (b) Event Frame details in PI Vision; (c) notification rule sent to e-mail.

On the left, the outlet molar flow and its compositions are depicted, providing a monitoring of reactant consumption and product forming. Acetone and H_2_ trends show a peak when the temperature approximates 375 °C and then sink when it is above 390 °C, proving that the studied optimized temperature is accurate. They help understand whether the inlet flows are adequate and react accordingly to the amount of product being generated. This may help identify issues, e.g., valve opening, raw material’s purity, reactor level, catalyst activity, and the effects of temperature in the reaction. In the middle, a trend of the temperature control with the safe operating limits, from 320 to 415 °C (red dashed lines), is displayed, accompanied by a multistate bar, which indicates if the temperature is within that range (green) or how far out (yellow and red). Heat duty and reaction rate are side by side for a closer comparison of the influence of the former to that of the latter. They help with evaluating the temperature effect on reaction rate, also by double-checking if the reactor is operating within safety limits from the wall material, raw materials, and product, avoiding their thermal degradation temperature. The inlet properties, such as temperature, pressure, and inlet molar flow rates, which are crucial to the proper reaction occurring, are also monitored. On the right side, a utility comparison is conducted, calculating sustainability and economic indicators to allow real-time evaluation of a process condition. Those variables are fuel cost rate, CO_2_ emission, fuel mass flow, and heat loss. On the biomass side, it is noted that its fuel cost rate is lower than FH1000s; it does not emit CO_2_, while FH1000 emits at least 153 kg/h; it consumes less fuel than FH1000 (around 3%); and therefore, it loses less heat than FH1000 in the same proportion.

Lastly, an Event Frame table, containing the Event Frame (alert) that occurred during the displayed time frame, with its name, asset (R-1101), start and end times, and severity, displays all the process anomalies that occurred or are occurring within the dashboard time frame. It is possible to visit this alert by clicking on it and having a deeper look at the variable trend, especially prior to the process anomaly. Also, a notification would be sent to the operator’s e-mail if a process anomaly occurs.

In this case, the anomaly is noted by the increase of fuel mass flow and its exceeding of the high operating limit, therefore raising the reactor’s temperature (outlet temperature) and, consequently, heat duty. Then, an Event Frame (alert) is generated and is shown as in Figure 6b, showing the behavior of related variables that can be added to the trend in the last 30 min prior to the event, which could help identify the root cause of the problem, e.g., heat duty, fuel mass flow (from each utility), inlet pressure, inlet molar flow, and reaction rate. An e-mail, containing information and a hyperlink to further details on PI Vision about the event, was generated and alerted the responsible personnel as well, as shown in Figure 6c. Now, the reason behind this rise must be further investigated by the company, e.g., failure or malfunctioning in a controller system, an operator manually opening a valve, and others.

Overall, this dashboard allows plant personnel to monitor the main variables and KPIs in the reaction section. Depending on the client, different KPIs are interesting to be monitored. The chosen and developed ones in this work aim at both the operational personnel and management, since the target public may vary. As seen in Figure 6a, several KPIs can be monitored, such as outlet molar flows from the reactor, reaction temperature, reaction duty and rate, inlet properties (pressure, temperature, and molar flows), and utility comparison (price, CO_2_ mass emission, mass flow, and heat loss, based on the type of utility chosen).

The PI Vision dashboard allows the analyses by the operational personnel on how the process behaves when a variable changes, e.g., how is the product molar flow rate considered in the raw material inlet molar flow rate? Is the raw material pure enough? Should we change the suppliers? If the reactor temperature increases, then how does the reaction rate respond? What is the cost of each fuel? Which one is cheaper at the moment (prices can fluctuate over time)? How much heat is lost with this fuel source? How is the process impacting the environment by using this fuel source? If a process anomaly occurs (shown in the Event Frame table: name, start time, duration, and severity), one can click on it, observe the related variables, and identify the sources of the problem, correcting it. These questions are examples of what can be considered when analyzing the dashboard and may help with choosing raw material sources, fuel sources, providing maintenance, and monitoring process health and safety.

## Conclusions

In the present work, the production process of acetone via IPA dehydrogenation was simulated, and alternative scenarios were proposed to improve economic indicators, hence reducing costs and/or increasing production in an eco-friendly way, as an example of how process simulation coupled with data historians and analyzers could be applied to enhance process sustainability. The simulations were carried out in the Aspen Plus v.12.1 process simulator. Possessing simulated data, pieces of equipment were designed, and economic and sustainability evaluations were conducted. A PI Asset Framework structure, a PI Vision dashboard, and one Event Frame with e-mail notification were developed to provide process monitoring and deepen plant personnel analysis. The purpose was to compare and confirm economic and sustainable viabilities of the process and its different scenarios and to simulate an operator monitoring the process daily, mainly if a change occurs.

Conventional and biomass scenarios were profitable but not economically feasible due to high TPC, with IPA being the main factor responsible for increasing operational costs. It was necessary to create alternatives, to adapt, and to cut expenses to make it economically feasible, such as reaction temperature optimization or utility change, to obtain a positive NPV. In these alternative scenarios, it is noted that even by increasing operational costs, one can enhance its revenues and overcome costs without overlooking environmental safety and metrics.

In relation to all sustainability and economic metrics, the TOpt biomass scenario is the most attractive and advantageous scenario. This is the most interesting one since it combined the cost reduction of heating utilities, a higher production of acetone, even if it meant a higher use of the reactor’s fuel but within the acceptable economic and safety boundaries, and the lowest impact on the environment. The process’ revenues increased by 5.2%, its production costs reduced by 0.26%, it achieved a NPV of US$4.63 M (372% higher than the baseline), and it was six times more sustainable than the conventional scenario. Finally, this scenario made the investment economically viable and feasible during the established project horizon and within five years of operation.

It was demonstrated how the PI System is able to provide insights into what scenarios are best in comparison to others in an online dashboard. This helps plant personnel in decision-making, giving an overview of the process with the necessary preliminary data. Receiving process anomaly alerts in real time via e-mail reduces the response time and speeds up correction, also providing possible root causes for them and helping with their identification. Unified and automated data and reports are available for the whole company without the need to manually share individual spreadsheets. Therefore, one can closely monitor process operation and modifications, evaluating and reporting if the plant is safely adapting to them while observing economic and sustainability metrics as key process indicators. The novelty of this work lies in exploiting the true potential of a data historian and promoting it from the state of a simple database to what it really is: a data analyzer.

## Supplementary Material



## Notes

Complete description of the simulations performed in Aspen Plus and estimated production costs and stream properties (PDF)

## Abbreviations

CAPEX: capital expenditure
CI: carbon intensity
CW: cooling water
CF: criticality factor
D/F: distillate-to-feed ratio
EF: *E*-factor
FH1000: fired heat 1000
FOB: free on board
GDC: green design criteria
HEX: heat exchanger
HETP: height equivalent to a theoretical plate
HPS: high-pressure steam
HHV: higher heating value
IPA: isopropyl alcohol
KPI: key process indicator
LPS: low-pressure steam
NPV: net present value
NRTL: non-random two-liquid
NV: normalized value
OPEX: operating expenditure
cref: reference criticality factor
RR: reflux ratio
CHILLED: river water
SQM: scoring quantitative metric
SI: severity index
SM: severity matrix
SRK: Soave–Redlich–Kwong
SD: sustainability degree
TOpt: temperature optimization (optimized temperature)
TI: thermal intensity
TPC: total production costs
UNIQUAC: Universal Quasichemical
WI: water intensity
