# Supplementary material for: Technical, Economic, and Sustainability Evaluation Integrated with PI System Monitoring: A Case Study on Different Process Alternatives for Acetone Production
Source: ACS Omega. 2025 Jul 23;10(31):34372–88. doi: 10.1021/acsomega.5c02293 (PMC12355327; doi:10.1021/acsomega.5c02293)
Supplement: Supplementary file 1 [file ao5c02293_si_001.pdf]

Supplementary Material for "Technical, Economic and Sustainability Evaluation Integrated with PI System Monitoring: Case Study on Different Process Alternatives for Acetone Production"

Matheus Vittori Ferreira [1*], André Ferreira Young [2] and José Carlos Costa da Silva Pinto [1]

[1] Escola de Química, Universidade Federal do Rio de Janeiro, Av. Athos da Silveira Ramos, 149, Rio de Janeiro, Brazil.

[2] Departamento de Engenharia Química e de Petróleo, Universidade Federal Fluminense, Rua Passo da Pátria, 156, Niterói, Brazil.

[*] matheusvittori@eq.ufrj.br

Contents

This document presents:

- 16 pages;
- 17 tables.
- 1 Figure.

It is important to state that utilized capital cost formulas are valid for a range of a respective dimension, e.g., HEX area between 2 ft^2 and 200 ft^2 for Double Pipe types and 150 ft^2 and 12,000 ft^2 for the other ones, pump volumetric flow rate between 250 gpm and 5,000 gpm depending on the characteristics of the chosen pump (e.g., flow rate range for centrifugal pump, with one stage, 1,800 shaft rpm and horizontal split case) etc. Therefore, in some cases when a piece of equipment did not present the respective dimension within those limits, the boundary value would be used instead, so it fits within the valid range.

E-1101, E-1103, E-1106, and E-1107 were not Double Pipe and their areas were smaller than 150 ft^2, but were considered to be equal to this limit for a conservative approach. Estimated values were considered for the global heat transfer coefficients according to Seider [2]. The employed utilities for the processes were high- and low-pressure steams for heating [18], at a temperature of 254 °C and 135 oC, respectively, cooling and river waters for cooling, at 30 °C and 7.22 ºC, respectively, and Fired Heat 1000 (FH1000) at 1000 °C for the reactor's fuel (in substitution to the saline solution circulation, as explained).

R-1101 is a heated reactor. Therefore, the fired heater cost formula was used. Although its heat exchanged is below the formula valid range, it was possible to linearly extrapolate the correlation to that value without inconsistencies.

P-1104A/B volumetric flow rate was smaller than 250 gpm, and needed to be considered equal to this limit, due to the formula valid range.

Table S1. Process streams of the Conventional and Biomass scenarios.

Stream	Units	ST-1	ST-2	ST-3	ST-5	ST-6
Vapor Faction		0	0	1	1	0
Temperature	°C	25	32.2	350	20	37.6
Pressure	kPa	1	2.3	2.2	1.6	1.5
Mass Enthalpy	cal/g	-1586.7	-1665.3	-1092.4	-650.2	-2930.6
Volume Flow	L/min	45.8	50.7	36969.6	8918	10.2
Molar Flow	kmol/h	52	59.6	92.7	35.8	21.6
Mass Flow	kg/h	2401.3	2587	2587	219.6	492.7
Mass Fractions						
IPA		0.871	0.809	0.040	0.001	0.000
Acetone		0.000	0.027	0.770	0.686	0.306
H2		0.000	0.000	0.026	0.304	0.000
Water		0.129	0.164	0.164	0.010	0.694
**Stream**	**Units**	**ST-7**	**ST-8**	**ST-9**	**ST-11**	**ST-12**
Vapor Faction		1	0	0	0	0
Temperature	°C	32.7	25	23.9	56	112.9
Pressure	kPa	1.5	2	1.5	1.2	1.4
Mass Enthalpy	cal/g	-733.2	-3788.8	-1769.4	-1001.8	-2635.3
Volume Flow	L/min	9671.7	6.8	64.3	39.8	29.4
Molar Flow	kmol/h	34.2	20	78.5	27.4	51
Mass Flow	kg/h	87.2	360.3	2860.1	1589.4	1265.4
Mass Fractions						
IPA		0.000	0.000	0.036	0.000	0.082
Acetone		0.000	0.000	0.696	1.000	0.313
H2		0.765	0.000	0.000	0.000	0.000
Water		0.235	1.000	0.267	0.000	0.605

Table S1. Process streams of the Conventional and Biomass scenarios (continuation).

Stream	Units	ST-14	ST-15	ST-16	E-1101-O	E-1102-O
Vapor Faction		0	0	1	1	0.44
Temperature	°C	107.1	113	32.9	234	45
Pressure	kPa	1.2	1.4	1.2	2.2	1.8
Mass Enthalpy	cal/g	-2683.2	-2627.8	-741.5	-1332.6	-1422.2
Volume Flow	L/min	4.3	25.1	12131.7	19141.5	10127.1
Molar Flow	kmol/h	7.7	43.4	34.3	59.6	92.7
Mass Flow	kg/h	185.7	1079.7	92.5	2587	2587
Mass Fractions						
IPA		0.000	0.096	0.000	0.809	0.04
Acetone		0.373	0.303	0.057	0.027	0.77
H2		0.000	0.000	0.721	0	0.026
Water		0.627	0.601	0.222	0.164	0.164
**Stream**	**Units**	**E-1103-O**	**E-1108-O**	**T-1102-T**	**T-1103-I**	**M-1101-O**
Vapor Faction		0.39	0	1	0	0
Temperature	°C	20	45	56	113	32.1
Pressure	kPa	1.6	1.3	1.2	3	1
Mass Enthalpy	cal/g	-1453.2	-2689.2	-877	-2635.2	-1665.4
Volume Flow	L/min	8971.6	22.8	38.9	29.4	50.7
Molar Flow	kmol/h	92.7	43.4	0.1	51	59.6
Mass Flow	kg/h	2587	1079.7	5.3	1265.4	2587
Mass Fractions						
IPA		0.04	0.096	0.000	0.082	0.809
Acetone		0.77	0.303	0.995	0.313	0.027
H2		0.026	0	0.005	0.000	0.000
Water		0.164	0.601	0.000	0.605	0.164

Table S1. Process streams of the Conventional and Biomass scenarios (continuation).

Stream	Units	V-1102-B
Vapor Faction		0
Temperature	°C	20
Pressure	kPa	1.6
Mass Enthalpy	cal/g	-1527.7
Volume Flow	L/min	53.6
Molar Flow	kmol/h	56.9
Mass Flow	kg/h	2367.4
Mass Fractions		
IPA		0.044
Acetone		0.778
H2		0.000
Water		0.179

Table S2. Process streams of the TOpt Conventional and TOpt Biomass scenarios.

Stream	Units	ST-1	ST-2	ST-3	ST-5	ST-6
Vapor Faction		0	0	1	1	0
Temperature	°C	25	31.9	375.5	20	38.9
Pressure	kPa	1	2.3	2.2	1.6	1.5
Mass Enthalpy	cal/g	-1586.7	-1664.8	-1068.3	-664.5	-2842.9
Volume Flow	L/min	45.8	50.4	38983	9369.2	10.7
Molar Flow	kmol/h	52	59.3	93.9	37.6	21.8
Mass Flow	kg/h	2401.3	2577.4	2577.4	244.4	511.9
Mass Fractions						
IPA		0.871	0.812	0.007	0.000	0.000
Acetone		0.000	0.024	0.802	0.706	0.337
H2		0.000	0.000	0.027	0.285	0.000
Water		0.129	0.164	0.164	0.009	0.663
**Stream**	**Units**	**ST-7**	**ST-8**	**ST-9**	**ST-11**	**ST-12**
Vapor Faction		1	0	0	0	0
Temperature	°C	34.2	25	24.4	56	112.1
Pressure	kPa	1.5	2	1.5	1.2	1.4
Mass Enthalpy	cal/g	-778.1	-3788.8	-1760.3	-1001.8	-2745.3
Volume Flow	L/min	10167.4	6.8	64.4	41.9	27
Molar Flow	kmol/h	35.8	20	78.1	28.8	49.2
Mass Flow	kg/h	92.8	360.3	2844.9	1671.4	1168.2
Mass Fractions						
IPA		0.000	0.000	0.006	0.000	0.015
Acetone		0.000	0.000	0.727	1.000	0.335
H2		0.750	0.000	0.000	0.000	0.000
Water		0.250	1.000	0.267	0.000	0.650

Table S2. Process streams of the TOpt Conventional and TOpt Biomass scenarios (continuation).

Stream	Units	ST-14	ST-15	ST-16	E-1101-O	E-1102-O
Vapor Faction		0	0	1	1	0.46
Temperature	°C	107.2	112.2	34.3	234	45
Pressure	kPa	1.2	1.4	1.2	2.2	1.8
Mass Enthalpy	cal/g	-2731.3	-2748.6	-783.4	-1332	-1408.4
Volume Flow	L/min	4	22.9	12750.6	19052.7	10749.2
Molar Flow	kmol/h	7.4	41.8	35.9	59.3	93.9
Mass Flow	kg/h	176.1	992.1	98.1	2577.4	2577.4
Mass Fractions						
IPA		0.000	0.018	0.000	0.812	0.007
Acetone		0.356	0.331	0.054	0.024	0.802
H2		0.000	0.000	0.710	0	0.027
Water		0.644	0.651	0.237	0.164	0.164
**Stream**	**Units**	**E-1103-O**	**E-1108-O**	**T-1102-T**	**T-1103-I**	**M-1101-O**
Vapor Faction		0.4	0	1	0	0
Temperature	°C	20	45	56	112.3	31.8
Pressure	kPa	1.6	1.3	1.2	3	1
Mass Enthalpy	cal/g	-1441.3	-2809.3	-877	-2745.1	-1664.9
Volume Flow	L/min	9422.4	20.9	38.5	27	50.4
Molar Flow	kmol/h	93.9	41.8	0.1	49.2	59.3
Mass Flow	kg/h	2577.4	992.1	5.3	1168.2	2577.4
Mass Fractions						
IPA		0.007	0.018	0.000	0.015	0.812
Acetone		0.802	0.331	0.995	0.335	0.024
H2		0.027	0	0.005	0.000	0.000
Water		0.164	0.601	0.000	0.605	0.164

Table S2. Process streams of the TOpt Conventional and TOpt Biomass scenarios (continuation).

Stream	Units	V-1102-B
Vapor Faction		0
Temperature	°C	20
Pressure	kPa	1.6
Mass Enthalpy	cal/g	-1522.7
Volume Flow	L/min	53.2
Molar Flow	kmol/h	56.3
Mass Flow	kg/h	2333
Mass Fractions		
IPA		0.007
Acetone		0.813
H2		0.000
Water		0.180

Table S3. Heat exchangers of the Conventional and Biomass scenarios.

Equipment	Duty (J/s)	Area (m2)	U (BTU/(°F-h-ft2))	Utility Flow (kg/h)	Cost (US$)
E-1101	1000756	9.15	250.00	2095.43	28545.15
E-1102	-992228	14.94	100.00	171057.3	28620.32
E-1103	-93174.1	25.68	50.00	3210.92	30096.78
E-1104 (Condenser T-1102)	-358410	17.74	100.00	61788.88	15110.09
E-1105 (Reboiler T-1102)	508534.9	30.38	250.00	835.23	30899.45
E-1106 (Condenser T-1103)	-423339	8.79	100.00	72982.46	14788.45
E-1107 (Reboiler T-1102)	422182.3	25.35	250.00	693.4	30041.1
E-1108	-77089.1	2.71	100.00	13289.93	3438.29

Table S4. Pumps of the Conventional and Biomass scenarios.

Equipment	Volume Flow (gal/min)	Pressure Increase (bar)	Power (HP)	Utility Usage (kW)	Cost (US$)
P-1101	250.00	1.29	1.00	0.27	17841.7
P-1104	250.00	1.6	1.00	0.2	18435.14

Table S5. Reactor of the Conventional and Biomass scenarios.

Equipment	Number of Vessels	Number of Tubes	Tube Length (m)	Tube Diameter (m)	Cost (US$)
R-1101	1	448	6.096	0.0508	211722.7

Table S6. Vessel of the Conventional and Biomass scenarios.

Equipment	Number of Vessels	Length / Diameter (m)	Cost (US$)
V-1102	1	1.60 / 0.65	13164.70

Table S7. Towers of the Conventional and Biomass scenarios.

Equipment	Top Temperature (°C)	Bottoms Temperature (°C)	Top Pressure (kPa)	Bottoms Pressure	Height (m)
T-1101	32.73	37.57	1.5	1.5	3.2
T-1102	56	112.88	1.2	1.4	37
T-1103	107.14	113.05	1.2	1.4	17.96

Equipment	Diameter (m)	Number of stages	Reflux Ratio (molar)	Cost (US$)
T-1101	0.28	7	-	62536.97
T-1102	0.58	66	0.6	137920.19
T-1103	0.45	19	4.246	64308.14

Table S8. Heat exchangers of the TOpt Conventional and TOpt Biomass scenarios.

Equipment	Duty (J/s)	Area (m2)	U (BTU/(°F-h-ft2))	Utility Flow (kg/h)	Cost (US$)
E-1101	997526.91	9.11	250.00	2088.67	28545.15
E-1102	-1019635.82	14.57	100.00	175782.21	28590.08
E-1103	-98634.7	27.19	50.00	3399.1	30349.39
E-1104 (Condenser T-1102)	-376882.05	18.65	100.00	64973.35	15199.89
E-1105 (Reboiler T-1102)	518469.76	29.44	250.00	851.55	30735.28
E-1106 (Condenser T-1103)	-409517.43	8.5	100.00	70599.6	14788.45
E-1107 (Reboiler T-1102)	408417.82	23.06	250.00	670.8	29672.25
E-1108	-70041.34	2.48	100.00	12074.92	3389.2

Table S9. Pumps of the TOpt Conventional and TOpt Biomass scenarios.

Equipment	Volume Flow (gal/min)	Pressure Increase (bar)	Power (HP)	Utility Usage (kW)	Cost (US$)
P-1101	250.00	1.29	1.00	0.27	17839.84
P-1104	250.00	1.6	1.00	0.18	18423.04

Table S10. Reactor of the TOpt Conventional and TOpt Biomass scenarios.

Equipment	Number of Vessels	Number of Tubes	Tube Length (m)	Tube Diameter (m)	Cost (US$)
R-1101	1	448	6.096	0.0508	227185.15

Table S11. Vessel of the TOpt Conventional and TOpt Biomass scenarios.

Equipment	Number of Vessels	Length / Diameter (m)	Cost (US$)
V-1102	1	1.47 / 0.68	13010.29

Table S12. Towers of the TOpt Conventional and TOpt Biomass scenarios.

Equipment	Top Temperature (°C)	Bottoms Temperature (°C)	Top Pressure (kPa)	Bottoms Pressure (kPa)	Height (m)
T-1101	34.15	38.88	1.5	1.5	3.2
T-1102	56	112.15	1.2	1.4	37
T-1103	107.24	112.17	1.2	1.4	17.96

Equipment	Diameter (m)	Number of stages	Reflux Ratio (molar)	Cost (US$)
T-1101	0.28	7	-	62663.33
T-1102	0.59	66	0.6	138832.37
T-1103	0.44	19	4.246	63719.98

Table S13. SQM grades of each scenario (NM).

Scenario	CI Grade	EF Grade	TI Grade	WI Grade
Conventional	3	3	3	3
Biomass	1	3	3	1
TOpt Conventional	3	1	1	3
TOpt Biomass	1	1	1	1

Table S14. GDC grades of each scenario.

Scenario	GDC 01	GDC 02	GDC 03	GDC 04	GDC 05	GDC 06	GDC 07
Conventional	1.0	1.0	1.0	3.0	9.0	1.0	1.0
Biomass	1.0	1.0	1.0	1.0	1.0	1.0	1.0
TOpt Conventional	1.0	1.0	1.0	9.0	9.0	1.0	1.0
TOpt Biomass	1.0	1.0	1.0	1.0	1.0	1.0	1.0

Scenario	GDC 08	GDC 09	GDC 10	GDC 11	GDC 12	GDC 13	GDC 14
Conventional	1.0	0.0	0.0	1.0	1.0	0.0	0.0
Biomass	1.0	0.0	0.0	1.0	1.0	0.0	0.0
TOpt Conventional	1.0	0.0	0.0	1.0	1.0	0.0	0.0
TOpt Biomass	1.0	0.0	0.0	1.0	1.0	0.0	0.0

Table S15. Means of GDC grades of each scenario.

Scenario	Mean
Conventional	2.0
Biomass	1.0
TOpt Conventional	2.6
TOpt Biomass	1.0

Table S16. Multi-criteria severity matrix (SM).

Scenario	CI Grade	EF Grade	TI Grade	WI Grade
Conventional	6.0	6.0	6.0	6.0
Biomass	3.0	1.0	3.0	1.0
TOpt Conventional	2.6	7.8	2.6	7.8
TOpt Biomass	1	1	1	1

Table S17. Process streams of the reference work of Turton et al. and their deviations to the simulated process.

Stream	Turton et al. ST-1	ST-1 Deviation	Turton et al. ST-2	ST-2 Deviation
Vapor Faction	0	0%	0	0%
Temperature	25 °C	0%	32,2 °C	0%
Pressure	1.01 kPa	-1%	2.3 kPa	0%
Molar Flow	51.96 kmol/h	0%	57.84 kmol/h	3%
Component Molar Flow				
IPA	34.820 kmol/h	0%	38.640 kmol/h	-10%
Acetone	0.000 kmol/h	0%	0.160 kmol/h	646%
H2	0.000 kmol/h	0%	0.000 kmol/h	0%
Water	17.140 kmol/h	0%	19.040 kmol/h	24%
**Stream**	**Turton et al. ST-3**	**ST-3 Deviation**	**Turton et al. ST-5**	**ST-5 Deviation**
Vapor Faction	1	0%	1	0%
Temperature	350 °C	0%	20 °C	0%
Pressure	1.91 kPa	15%	1.6 kPa	0%
Molar Flow	92.62 kmol/h	0%	39.74 kmol/h	-10%
Component Molar Flow				
IPA	3.860 kmol/h	-55%	0.120 kmol/h	-98%
Acetone	34.940 kmol/h	-2%	4.440 kmol/h	-42%
H2	34.780 kmol/h	-5%	34.780 kmol/h	-5%
Water	19.040 kmol/h	24%	0.400 kmol/h	-69%

Table S17. Process streams of the reference work of Turton et al. and their deviations to the simulated process (continuation).

Stream	Turton et al. ST-6	ST-6 Deviation (%)	Turton et al. ST-7	ST-7 Deviation (%)
Vapor Faction	0	0%	1	0%
Temperature	27 °C	39%	33 °C	1%
Pressure	1.6 kPa	-6%	1.5 kPa	0%
Molar Flow	21.14 kmol/h	2%	38.6 kmol/h	11%
Component Molar Flow				
IPA	0.100 kmol/h	-98%	0.020 kmol/h	100%
Acetone	1.930 kmol/h	34%	2.510 kmol/h	100%
H2	0.000 kmol/h	0%	34.780 kmol/h	5%
Water	19.110 kmol/h	-1%	1.290 kmol/h	12%
**Stream**	**Turton et al. ST-8**	**ST-8 Deviation (%)**	**Turton et al. ST-9**	**ST-9 Deviation (%)**
Vapor Faction	0	0	0	0%
Temperature	25 °C	0	22 °C	-9%
Pressure	2 kPa	0	1.6 kPa	6%
Molar Flow	20 kmol/h	0	74.02 kmol/h	-6%
Component Molar Flow				
IPA	0.000 kmol/h	0	3.840 kmol/h	55%
Acetone	0.000 kmol/h	0	32.430 kmol/h	-6%
H2	0.000 kmol/h	0	0.000 kmol/h	0%
Water	20.000 kmol/h	0	37.750 kmol/h	-12%

Table S17. Process streams of the reference work of Turton et al. and their deviations to the simulated process (continuation).

Stream	Turton et al. ST-11	ST-11 Deviation (%)	Turton et al. ST-12	ST-12 Deviation (%)
Vapor Faction	0	0%	0	0%
Temperature	61 °C	8%	90 °C	-25%
Pressure	1.5 kPa	20%	1.4 kPa	0%
Molar Flow	32.29 kmol/h	15%	41.73 kmol/h	-22%
Component Molar Flow				
IPA	0.020 kmol/h	100%	3.820 kmol/h	55%
Acetone	32.270 kmol/h	15%	0.160 kmol/h	-4169%
H2	0.000 kmol/h	0%	0.000 kmol/h	0%
Water	0.000 kmol/h	0%	37.750 kmol/h	-12%
**Stream**	**Turton et al. ST-14**	**ST-14 Deviation (%)**	**Turton et al. ST-15**	**ST-15 Deviation (%)**
Vapor Faction	1	100%	0	0%
Temperature	83 °C	-29%	109 °C	-4%
Pressure	1.2 kPa	0%	1.4 kPa	0%
Molar Flow	5.88 kmol/h	-31%	35.85 kmol/h	-21%
Component Molar Flow				
IPA	3.820 kmol/h	100%	0.000 kmol/h	0%
Acetone	0.160 kmol/h	-646%	0.000 kmol/h	0%
H2	0.000 kmol/h	0%	0.000 kmol/h	0%
Water	1.900 kmol/h	-240%	35.850 kmol/h	0%

Table S17. Process streams of the reference work of Turton et al. and their deviations to the simulated process (continuation).

Stream	Turton et al. ST-16	ST-16 Deviation (%)
Vapor Faction	0	0%
Temperature	33 °C	0%
Pressure	1.2 kPa	0%
Molar Flow	38.6 kmol/h	11%
Component Molar Flow		
IPA	0.020 kmol/h	100%
Acetone	2.510 kmol/h	96%
H2	34.780 kmol/h	5%
Water	1.290 kmol/h	12%

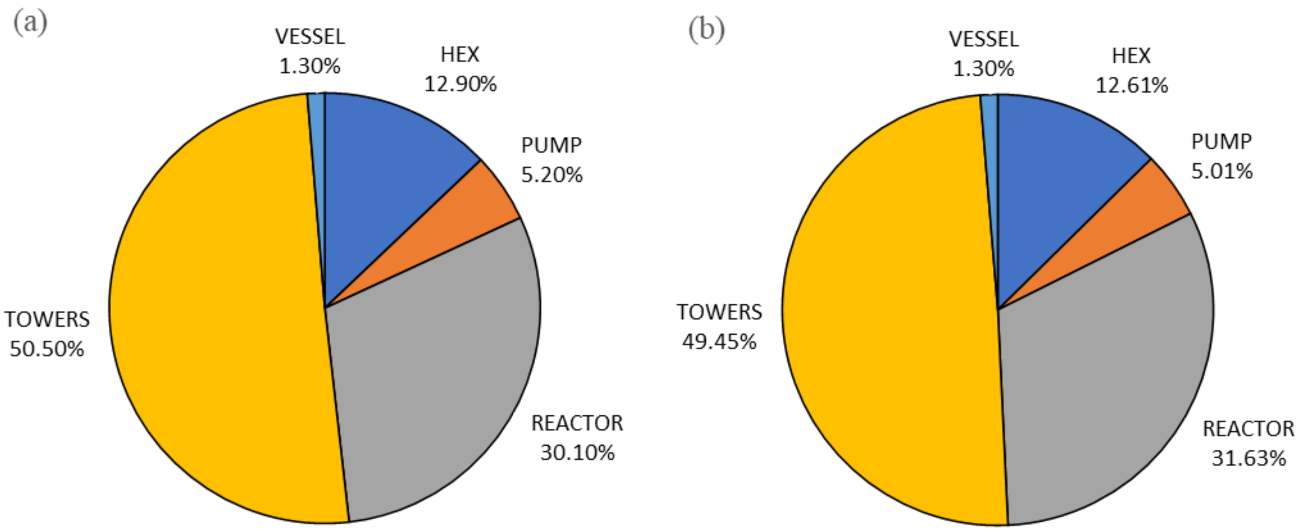

Figure S1. Equipment cost distribution for the: (a) Conventional and Biomass routes; (b) TOpt Conventional and TOpt Biomass routes.
